# Assessment of the Core Competencies for Disaster Management Among Nurses Dealing With Casualties: A Study From Yemen

**DOI:** 10.7759/cureus.78834

**Published:** 2025-02-10

**Authors:** Mohammed M Al-Jabri, Adel Aljawfi, Marzoq A Odhah, Khalaf A Alshammari, Samah Zidan, Bashair Abdo, Omar Abdelwahab, Sherif Mohamed

**Affiliations:** 1 Nursing Science, Prince Sattam bin Abdulaziz University, Wadi Aldawaser, SAU; 2 Family and Community Health Nursing, Faculty of Medical Sciences, Thamar University, Dhamar, YEM; 3 Nursing and Midwifery, Al Razi University, Sanaa, YEM; 4 Critical Care Nursing, Sanaa University, Sanaa, YEM; 5 Respiratory Care, Al Razi University, Sanaa, YEM; 6 Critical Care, King Salman Specialist Hospital, Hail, SAU; 7 Adult Health Nursing, School of Nursing, Badr University, Cairo, EGY; 8 Nursing Administration, Faculty of Nursing, Damanhour University, Damanhour, EGY; 9 Medicine, Alfaisal University, Riyadh, SAU; 10 Internal Medicine, Sultan Bin Abdelaziz Humantarian City, Riyadh, SAU; 11 Chest Diseases and Tuberculosis, Faculty of Medicine, Assiut University, Assiut, EGY

**Keywords:** competency-based assessment, critical care nurse, emergency nurse, feasibility assessment, nurse managers, professional nurse, registered nurse

## Abstract

Background

Yemen has faced numerous war-related disasters in recent years, yet limited research exists on the disaster core competencies and preparedness of Yemeni nurses. This study evaluated the disaster core competencies and preparedness levels of Yemeni nurses managing disasters.

Methods

A cross-sectional descriptive study. Data were collected from 126 nurses using a demographic questionnaire and the Nurses' Perceptions of Disaster Core Competencies (NPDCC) scale. Inferential statistics, including independent t-tests and one-way ANOVA, assessed relationships between demographic characteristics and competency levels. Data normality was verified using the Kolmogorov-Smirnov test.

Results

The mean competency score was moderate (3.43 ± 0.18). General diagnostic skills scored the highest (4.01 ± 0.12), while special diagnostic skills scored the lowest (2.98 ± 0.44). Nurses with master’s degrees (t = 4.3, p = 0.010), those with work experience in previous incidents and disasters (t = 2.9, p = 0.032), those attending disaster training course/drill (t = 3.7, p = 0.005), and those with workplaces prepared with an established disaster plan (t = 4.6, p = 0.002) had significantly higher scores for competencies.

Conclusions

In the current study, Yemeni nurses demonstrated moderate competencies in disaster risk management, with gaps in specialized diagnostic skills. Nurses with master’s degrees, those with work experience in previous incidents and disasters, those attending the disaster risk management training course/drill, those with more extended professional experience, and those with workplaces prepared with an established disaster plan had significantly higher scores in disaster risk management. Enhancing disaster preparedness should focus on targeted training, institutional planning, and resource allocation. These initiatives will strengthen the resilience of the nursing workforce in Yemen and similar conflict-affected regions.

## Introduction

The literature describes disaster nursing as the “systematic utilization of nursing skills and knowledge in disasters and the development of policies and practices to reduce disaster health consequences and eliminate its life-threatening hazards” [1,2]. Nurses play an important role as direct care providers and leaders during disasters, making their preparedness and competency essential for effective response [3].

Despite increasing recognition of the importance of disaster preparedness, many nurses remain underprepared and lack the necessary competencies to respond effectively. These competencies include different aspects related to emergency preparedness knowledge, risk assessment and triage skills, clinical and technical proficiency, psychological support and communication, cultural competence, proper leadership and teamwork, and adequate education and training [4-6].

Globally, studies have investigated nurses' roles, perceptions, and competencies in disaster risk management, often finding significant shortcomings in one or more core competency areas [6-8]. However, there is a notable scarcity of research on disaster nursing competencies in Arab countries [6,9], including Yemen. This gap is particularly concerning given Yemen's recent history of war-related accidents and disasters, which have placed immense demands on healthcare workers. Limited research on this topic hinders the development of targeted strategies to empower nurses and improve their disaster preparedness.

Yemen has been grappling with protracted armed conflicts that have had a devastating impact on its healthcare systems [10]. The ongoing conflicts have resulted in the widespread destruction of healthcare services, severely limited access to essential healthcare services, and critical shortages of medical supplies and personnel [10]. Understanding the specific challenges nurses face in conflict zones such as Yemen and ensuring they are adequately trained and supported is crucial for delivering effective healthcare services and building resilient health systems in these vulnerable regions [3,10].

The current study analyzes Yemeni nurses' disaster readiness and essential skills to pinpoint competency deficiencies and explore how their training background and work history affect their skill levels. By identifying strengths and areas for improvement, the findings will inform the development of effective training programs and policies to enhance the disaster management capabilities of nurses in Yemen and similar contexts.

## Materials and methods

Study design and setting

This cross-sectional descriptive study was conducted between July and December 2024 at two Yemeni University hospitals that treated disasters of armed conflicts in that area. These hospitals have experienced frequent presentations of casualties from armed conflicts at different times. The study population comprised all nurses who had treated casualties of the armed conflict.

Aims and objectives of the study

The study aims to evaluate Yemeni nurses' perceptions of their disaster preparedness and core competencies.

Study population and sampling

To recruit the nursing participants, we utilized a convenience sampling technique. The inclusion criteria were: first, being a nurse; second, having a minimum of 12 months experience in the same healthcare facility; and third, being willing electively to participate in this study. The reason beyond working in the same facility is to be familiar with that facility's policies and procedures. To determine the minimum sample size of the study’s participants, the authors calculated it based on the conventional criterion of statistical significance (α) of 0.05, a conventional power of 0.80, and a medium effect size using the G*Power 3.1.2 (Ziff Davis Inc., New York City, NY) and eight predictors. It was initially estimated for the sample size in this study to be 110. However, an additional 10% was added to account for incomplete questionnaires, thus yielding a total sample size of 120.

Data collection tools

Demographic Characteristics Questionnaire

Collected information on demographic features such as gender, age, professional experience, education level, work area, prior disaster experience, and attendance at disaster risk management training.

Questionnaire of the Nurses’ Perceptions of Disaster Core Competencies (NPDCC) Scale

The current study used a 45-item scale that includes five subscales: critical thinking skills, skills for special diagnostic, general diagnostic skills, technical skills, and communication skills. Each subclass contained a number of items. These numbers were four, six, 13, 14, and eight items, respectively. 

These 45 competency questions were explicitly developed for armed conflict zones, and one question asked for any comments or suggestions. The questionnaire was established on the premise of “What do nurses need to be able to do?" Each competency measures a critical task that nurses should be capable of performing in their roles, each signifying an aspect of practical ability and theoretical knowledge essential for their professional duties.

The scale is Likert-type, where each item is scored from one (which indicates that it needs to be taught) to five (which signifies; I can do and teach it), with the lowest and highest scores being 1 and 5, respectively. As expected, a higher score signifies nurses’ better perceptions of core competencies [8]. This scale was developed by Celik in Turkey to investigate nurses’ perceptions of core competencies dur­ing disasters. Another Turkish study confirmed the reliability of this original scale on dedicated nurses using internal consistency, with reported Cronbach’s alpha coefficients of 0.81-0.92 and 0.96 for the subscales and the whole scale, respectively [11].

Thus, a score of 1-2.33 was interpreted as poor competency, a score of 2.34-3.66 was considered moderate competency, and a score above 3.66 was interpreted as high competency [8,11]. The validity of the adopted questionnaire was established through a pilot testing process. Researchers with relevant expertise evaluated the questionnaire’s content and clarity and assessed whether its questions effectively measured the intended constructs. The positive feedback provided for the questionnaire indicated its relevance and comprehensibility.

Ethical considerations 

The relevant ethical committee of the Faculty of Nursing approved the present research (approval number 102-L), dated July 18, 2024. Individual participants provided written informed consent. The questionnaires were coded to ensure anonymity and confidentiality.

Data analysis

Descriptive statistics were used to summarize demographic data and competency scores. Inferential statistics, including independent t-tests and one-way ANOVA, assessed relationships between demographic characteristics and competency levels. Data normality was verified using the Kolmogorov-Smirnov test. Analyses were performed using SPSS version 26 (IBM Corp., Armonk, NY), with a significance level of p < 0.05.

## Results

Participant characteristics

One hundred twenty-six nurses participated in the study, with a mean age of 33 years (± 5.4). The majority were female (61%), held a bachelor’s degree (62%), and worked in medical and surgical wards (38%). Only 40% had prior disaster experience, and 34% had attended disaster preparedness training. Additionally, 37% reported that their workplace had an established disaster plan. Table 1 shows these characteristics.

**Table 1:** Demographic characteristics of the study nurses and the relationship to the score of competencies (n = 126) ^*^T: Independent t-test, ^#^p-values are significant at the level of ≤ 0.05 F: One-way analysis of variance

Variables	Number (%)	Mean ± SD	Statistic test^*^	P-value^#^
Age (years)				
<30	55 (44)	56.19±13.11	t = 1.1	0.081
≥30	71 (56)	55.10±17.09		
Gender				
Male	49 (39)	52.16±18.88	t = 0.74	0.072
Female	77 (61)	54.14±19.63		
Marital status				
Single	27 (22)	53.23±8.78	F = 1.9	0.064
Married	81 (64)	51.24±19.99		
Divorced/widow	18 (14)	49.11±22.02		
Educational degree			t = 4.3	0.010
Diploma	25 (20)	48.22±18.66		
Bachelor	78 (62)	52.21±19.73		
Master	23 (18)	55.51±12.93		
Professional experience (years)				
≤5	24 (19)	48.12±18.16	F = 2.7	0.042
6-10	87 (69)	51.02±24.11		
>10	15 (12)	54.14±17.72		
Work area				
Medical/surgical ward	48 (38)	47.22±18.55	F = 0.71	0.420
Operating room/theatre	31 (25)	50.19±16.77		
ICU	23 (18)	53.33±14.39		
ED	24 (19)	52.27±19.71		
Work experience in prior disaster				
Yes	50 (40)	51.09±23.21	t = 2.9	0.032
No	76 (60)	55.12±17.20		
Disaster preparedness training/drills				
Yes	43 (34)	52.22±24.19	t = 3.7	0.005
No	83 (66)	56.88±17.16		
Is there an established disaster plan in your workplace?				
Yes	47 (37)	54.80±22.36	t = 4.6	0.002
No	79 (63)	58.48±6.18		

Competency scores

The overall mean competency score was moderate (3.43 ± 0.18). Subscale mean scores were as follows: General diagnostic skills: 4.01 ± 0.12 (highest), critical thinking skills: 3.22 ± 0.21, technical skills: 3.82 ± 0.11, communication skills: 3.12 ± 0.02, and special diagnostic skills: 2.98 ± 0.44 (lowest). Competency levels were categorized as high (16.4%), moderate (66.5%), and poor (17.1%) (Table 2).

**Table 2:** Score of nurses’ competencies needed for disaster risk management

Skill	Mean ± SD
Skills of critical thinking	3.22 ± 0.21
Skills of special diagnostic	2.98 ± 0.44
General diagnostic skills	4.01 ± 0.12
Technical skills	3.82 ± 0.11
Skills of communication	3.12 ± 0.02
Total scale score	3.43 ± 0.18

Relations between sociodemographic characteristics and competencies scores

Nurses with master’s degrees, prior disaster experience, disaster training, and workplaces with established disaster plans scored significantly higher in competencies (p < 0.05). No significant relations existed between age, gender, marital status, work area, and reported core competencies (Table 1).

## Discussion

The current study addressed the Yemeni nurses’ perception of their disaster preparedness and core competencies. Yemen has experienced many war-related disasters, and dealing with casualties and war victims is crucial and a principal nearly daily practice for Yemeni nurses. To the best of our knowledge, the current study is the second study (after that of Mani et al. [12]) addressing the core competencies of Yemeni nurses dealing with armed conflict-related disasters. The authors used a validated questionnaire examining 47 different competencies for nursing in armed conflict zones, which were subsequently ranked to identify those that were valued most and least. Results revealed that the highest-ranked competencies focused on immediate life-saving interventions and personal safety. On the other hand, those involving broader disaster management, such as understanding organizational disaster plans, post-death care, and risk identification, ranked lower [12]. The authors stated that this may indicate a tendency to prioritize direct clinical care over strategic planning and long-term recovery in disaster nursing education. Worldwide studies investigated nurses' perceptions and knowledge of disaster risk management, and the surprising result was that nurses had notifiable shortcomings, at least in one of the subscales of the NPDCC [5-8,11-13].

The current study results showed that the total mean score of nurses' competencies was moderate. Moreover, two-thirds of the study participants had moderate competencies, while 16.4% and 17.1% had high and poor competencies in disaster risk management, respectively. These results agree with an interesting integrative review study that found that nurses obtained a low to moderate level of preparedness in disaster management in Arabian and Asian countries, and less than 25% had disaster core competencies [14]. Other studies from Western countries reported nurses’ insufficient competencies in disaster management [1,15]. On the other hand, the current study results are not concordant with those studies that reported that nurses' competencies in disaster risk management were above average [16].

Similarities and differences to other studies could be attributed to the differences in the studied population's sociodemographic characteristics, survey instruments, type of disaster, nursing subspecialties, and different geographic logistics. The results showed that special and general diagnostic skill subscales received the lowest and highest mean scores, respectively. Other studies obtained similar results [8,17].

Nurses with advanced degrees, prior disaster experience, and access to disaster preparedness training demonstrated significantly higher competency scores. These results are similar to those reported by previous studies worldwide [5-9,16,18], including studies from Iran [8], Jordan [6], Indonesia [19], and Poland [18].

Together, these results underscore the importance of continuing education, targeted training programs, and institutional disaster planning. Such measures enhance nurses’ readiness and confidence, enabling them to respond effectively in crises [1,20]. We observed a significant difference in competency scores according to the nurses’ professional experience. This agrees with Goki et al. [8] and others [19,21,22].

Implications and recommendations for practice

This study emphasizes the need for targeted interventions to address competency gaps, particularly in special diagnostic skills. Recommendations include integrating simulation-based training, enhancing access to disaster risk management courses, and fostering multidisciplinary collaboration. Policymakers should prioritize the development of comprehensive disaster preparedness plans and ensure their dissemination across healthcare institutions.

 Strengths and limitations

The current study has some strengths. It addresses a critical issue in a conflict-affected region, highlighting the importance of nurse preparedness in managing casualties. The cross-sectional design, convenience sampling, and use of validated tools (NPDCC scale) provide a robust framework for assessing competencies. We included various demographic factors, educational backgrounds, and training exposure, giving a multidimensional view of competency levels. Lastly, the manuscript compares Yemeni nurses' competencies with global standards, enhancing the broader relevance of the findings.

On the other hand, the study has some limitations. Being a single-center study and because of the lack of previous studies measuring levels of core disaster competencies in Yemen, it is not easy to assume the generalizability of Yemeni nurses’ competencies based only on the results of this study. While special diagnostic skills were highlighted as weak, the manuscript could investigate why this gap exists and how it manifests in practice.

## Conclusions

The findings highlight moderate disaster risk management competencies among Yemeni nurses, with notable gaps in special diagnostic skills. Nurses with master’s degrees, those with work experience in previous incidents and disasters, those attending the disaster risk management training course/drill, those with more extended professional experience, and those with workplaces prepared with an established disaster plan had significantly higher scores for competencies in disaster risk management.

Enhancing disaster preparedness should focus on targeted training, institutional planning, and resource allocation. These initiatives will strengthen the resilience of the nursing workforce in Yemen and similar conflict-affected regions.
